# Cutaneous PAN and Common Variable Immunodeficiency: what causes what?

**DOI:** 10.1186/1546-0096-9-S1-P80

**Published:** 2011-09-14

**Authors:** Ilaria Pagnini, Gabriele Simonini, Francesca Lippi, Chiara Azzari, Rolando Cimaz

**Affiliations:** 1 Department of Pediatrics, University of Florence, Italy

## Background

Cutaneous polyarteritis nodosa (c-PAN) is a necrotizing vasculitis of small and medium-sized vessels limited to the skin, characterized by the presence of subcutaneous nodular, painful, non purpuric lesions with or without livedo reticularis occurring predominantly in the lower extremities, with no systemic involvement. The cause of c-PAN is unknown: infectious agents in childhood and immunodeficiency in adults have been associated with the disease.

## Aim

To our knowledge the association of c-PAN and Common Variable Immunodeficiency (CVID) has not been reported so far.

## Case report

A 2 year-5 months old girl was referred for intermittent fever and cutaneous erythematous, painful nodular lesions on feet, ankles and pretibial regions. A skin biopsy showed a necrotizing non-granulomatous vasculitis. Diagnosis of cutaneous polyarteritis nodosa was made and treatment with methotrexate (MTX), prednisone and ibuprofen was started. One year later, due to insufficient response, MTX was switched to azathioprine. We obtained a good clinical control in 4 months, thus NSAIDs and steroids were progressively stopped. At an 8 months follow-up, laboratory test were all in the normal range, except for progressive hypogammaglobulinemia (IgA and IgM, 29.4 mg/dl; and 28 mg/dl; IgG= 571 mg/dl). Immunological tests showed: IgG2 deficiency (42 mg/dl) total white count at lower limits, with lymphopenia (21.6%); lymphocyte subpopulations showed deficiency of CD19+ B cells (3%, normal values: 6-25%) and a poor response to protein vaccines. Due to the 1-year persistent remission on therapy, AZA was tapered and stopped. After several months, hypogammaglobulinemia persisted, thus excluding a potential side effect related to AZA treatment. Six months later, due to a disease flare, AZA was restarted in addition to prednisone (1 mg/kg/die) and IVIG every 4 weeks (2 gr/kg). IVIG treatment was added in order to combine their anti-inflammatory effect with the CVID replacement therapy. At last follow-up her c-PAN is in clinical remission on AZA only. IVIG treatment is still needed for replacement (400 mg/kg monthly), due to her CVID. Of note, during her long term (6 years) follow-up in our Unit, she never developed any major infection.

## Conclusions

c-PAN and CVID are both due to immune system dysregulation, with not well known mechanisms. In our patient, immunosuppressive therapy could have induced a B-immunodeficiency, or could have uncovered a pre-existing immunodeficient status. However, a casual association or the possibility of c-PAN as onset of CVID cannot be excluded.

